# Malaria trends in Bobo-Dioulasso, Burkina Faso (2011–2024): a retrospective laboratory-based study with implications for malaria control

**DOI:** 10.1016/j.parepi.2026.e00551

**Published:** 2026-10-01

**Authors:** Mamoudou Cissé, Kiswendsida Thierry Guiguemdé, Issaka Zongo, Nwintièro Léandre Gilchrist Gané Dabiré, Alamissa Soulama, Somkéta Thomas Venceslas Sawadogo, Seydou Nakanabo Diallo, Adama Zida

**Affiliations:** a Institut Supérieur des Sciences de la Santé, Université Nazi BONI, 01 1091 Bobo-Dioulasso, Burkina Faso; b Laboratoire de Recherche, Centre MURAZ, 01 BP390 Bobo-Dioulasso, Burkina Faso; c Unité de Formation et de Recherche en Sciences de la Santé, Université Joseph KI-ZERBO, 03 BP 7021 Ouagadougou, Burkina Faso; d Institut de Recherche en Sciences de Santé, Centre National de la Recherche Scientifique et Technologique, 01 BP 545 Bobo-Dioulasso, Burkina Faso

**Keywords:** Malaria, Prevalence, Temporal trends, Seasonality, Risk factors, Urban area, Burkina Faso

## Abstract

**Background:**

Malaria remains a major public health concern in Burkina Faso. This study aimed to determine the prevalence, temporal and seasonal trends, and risk factors for malaria in the urban population of Bobo-Dioulasso from 2011 to 2024, a period encompassing major phases of malaria control implementation in the country.

**Methods:**

A retrospective cross-sectional study was conducted using data from thick and thin blood smears performed at the Parasitology-Mycology Laboratory of Centre MURAZ. Sociodemographic data, temporal data, and biological data were collected from laboratory records using a structured electronic data collection form. Univariate and multivariate analyses were used to identify risk factors of malaria.

**Results:**

Among 17,044 samples, the overall prevalence of malaria was 11.85%, with *Plasmodium falciparum* accounting for 97.52% of infections. Malaria prevalence showed marked seasonality, peaking between September and November. Individuals examined during the rainy season were 1.73 times more likely to be infected than those examined during the dry season (aOR = 1.73; 95% CI: 1.55–1.93). Over time, prevalence increased significantly from 5.53% in 2011 to 31.26% in 2024 (*p* < 0.001). The odds of malaria were higher during 2015–2019 (aOR = 1.18; 95% CI: 1.02–1.34) and 2020–2024 (aOR = 3.02; 95% CI: 2.64–3.46) compared with 2011–2014. Male participants (aOR = 1.29; 95% CI: 1.16–1.43) and those aged groups 5–15 years (aOR = 1.81; 95% CI: 1.56–2.10) and 16–26 years (aOR = 1.50; 95% CI: 1.31–1.71) also had higher odds of malaria compared with females and participants aged >26 years, respectively.

**Conclusion:**

These findings highlight a resurgence of urban malaria in Bobo-Dioulasso, with significantly higher odds of infection during 2015–2019 and 2020–2024 compared with 2011–2014. Strengthening epidemiological surveillance, expanding seasonal malaria chemoprevention to school-aged children, and promoting a multisectoral, integrated approach are crucial to sustain malaria control efforts in Burkina Faso.

## 1 Introduction

Malaria continues to be a major cause of morbidity and mortality in tropical regions and remains a significant public health challenge, particularly in sub-Saharan Africa. In 2024, global malaria cases reached an estimated 282 million, an increase of 9 million from 2023, largely driven by rises in the World Health Organization (WHO) African Region (WHO, 2025). Malaria incidence rose from 62.7 to 64.0 cases per 1000 population at risk between 2023 and 2024, suggesting a continued increase in transmission. Sub-Saharan Africa continues to bear the heaviest burden, accounting for approximately 94% of global malaria cases and 95% of malaria-related deaths (WHO, 2025).

Urban malaria in Africa exhibits distinct epidemiological features. Although traditionally considered a rural disease, malaria transmission persists in many urban settings, with prevalence typically ranging from 10% to 30%, predominantly driven by *Plasmodium (P.) falciparum* and, in some areas of East Africa, by *P. vivax* (Merga et al., 2025). Urban transmission is influenced by a combination of socioeconomic, environmental, and behavioral factors, including population density, mobility, housing conditions, proximity to breeding sites, and climatic factors, contributing to marked heterogeneity in malaria risk (Merga et al., 2025).

Burkina Faso remains one of the countries most heavily affected by malaria in sub-Saharan Africa (WHO, 2025). In 2024, the country accounted for approximately 3% of global malaria cases and 2.7% of malaria-related deaths. Malaria continued to be the leading cause of healthcare utilization (33.6%), hospital admissions (43.9%), and mortality (8.7%), particularly among children under five years of age and pregnant women (Ministère de la santé, 2025). Nationwide, more than 17 million suspected malaria cases were reported in 2024, with a test positivity rate of 62.2%, indicating sustained transmission despite the implementation of several large-scale control measures by the national malaria control program (NMCP). These measures include the widespread distribution of long-lasting insecticidal nets (LLINs), seasonal malaria chemoprevention (SMC), intermittent preventive treatment in pregnancy, and strengthened early diagnosis and case management (Ministère de la santé, 2021).

Despite sustained control efforts, malaria transmission persists in urban areas of Burkina Faso, particularly in Bobo-Dioulasso, where it remains a major public health concern. In 2024, malaria incidence was estimated at 358.45 per 1000 in the general population and 524.35 per 1000 among children under five years of age, while malaria prevalence among pregnant women was 19.79% in 2022 (Ministère de la santé, 2025; Cissé et al., 2025). Nevertheless, recent data on malaria prevalence and temporal trends in this urban setting are limited and outdated (Sirima, 2013). A malaria surveillance study previously conducted at Centre MURAZ in Bobo-Dioulasso covered the period from 2001 to 2010 (Sirima, 2013). Given the substantial time elapsed since that study and the evolving epidemiological context, updated evidence is needed. The period 2011–2024 encompasses major malaria control interventions implemented in Burkina Faso, including national LLIN distribution campaigns conducted in 2013, 2016, 2019, and 2022, as well as the introduction of SMC in 2014 and its nationwide scale-up from 2016. To address this gap, the present study analyzes a more recent and extended dataset, enabling a robust assessment of annual, monthly, and seasonal patterns of malaria in an urban setting. This study aimed to determine malaria prevalence, characterize temporal and seasonal trends, and identify risk factors of malaria in Bobo-Dioulasso, Burkina Faso, from 2011 to 2024.

## 2 Materials and methods

### 2.1 Study design and setting

This retrospective cross-sectional study was conducted in Bobo-Dioulasso (11.1649° N; 4.3052° W), located in western Burkina Faso, approximately 360 km from Ouagadougou, the administrative capital. Bobo-Dioulasso is the country's economic capital and second-largest urban center, with a population of 984,603 inhabitants in 2019, of whom 76.4% are under 35 years of age (INSD, Ouagadougou: Institut National de la Statistique et de la Démographie, 2020). The city has a South Sudanian climate characterized by two distinct seasons: a rainy season from May to October and a dry season from November to April. Annual rainfall ranges from 1000 to 1300 mm, and temperatures vary between 15 °C and 38 °C. These climatic conditions favor endemic malaria transmission, particularly during the rainy season.

The study was based on data collected at the Parasitology-Mycology Laboratory of Centre MURAZ, a reference research and diagnostic center that receives patients and laboratory samples from health facilities serving the urban and peri-urban populations of Bobo-Dioulasso. Urban features such as open drainage systems and inadequately managed wastewater create suitable breeding sites for *Anopheles* mosquitoes. The predominant vector of *Plasmodium* spp. is *Anopheles arabiensis,* with sporozoite infection rates of 4.2% in urban environments and 6.2% in peri-urban settings of the city (Dabiré et al., 2012).

Malaria continues to pose a significant public health burden in Bobo-Dioulasso, with incidence rates of 358.45 per 1000 in the general population and 524.35 per 1000 among children under five (Ministère de la santé, 2025). In 2022, malaria prevalence among pregnant women was estimated at 19.79% (Cissé et al., 2025).

### 2.2 Data source

Data were collected from the registers of the Parasitology-Mycology Laboratory at Centre MURAZ. The laboratory provides routine parasitological diagnostic services to urban and peri-urban populations of Bobo-Dioulasso and collaborates with the NMCP. Malaria diagnosis recorded in the registers was based on routine light microscopy of thick and thin blood smears performed by qualified laboratory personnel according to standard procedures.

### 2.3 Study population

The study population comprised all individuals who underwent a thick and thin blood smear examination at the Parasitology-Mycology Laboratory of the Centre MURAZ between 1 January 2011 and 31 December 2024, irrespective of age, sex, or reason for consultation. Records containing complete parasitological data, including the test result, date of examination, and registration number, were eligible for inclusion. Duplicate records identified during data entry and data cleaning were excluded from the analysis.

### 2.4 Sample size and sampling

An exhaustive sampling approach was adopted. The study included all thick and thin blood smear examinations conducted at the Parasitology-Mycology Laboratory of Centre MURAZ from 1 January 2011 to 31 December 2024, resulting in a dataset of 17,214 records.

### 2.5 Data collection

After obtaining authorization from the Technical Director of the Centre MURAZ, the registers of thick and thin blood smear examinations covering the period from 1 January 2011 to 31 December 2024 were reviewed. Sociodemographic data (age and sex), month and year of diagnosis, and biological data (malaria status, *Plasmodium* species, parasite stage, and parasite density) were subsequently collected using a structured electronic data collection form.

### 2.6 Data analysis

Data from the thick and thin blood smear registers were entered and cleaned using Microsoft Excel 2019 and subsequently analyzed using Stata version 12 (StataCorp, College Station, TX, USA). Figures were generated using Excel 2019.

Participants' age was categorized into four groups: <5 years, 5–15 years, 16–26 years, and > 26 years. Calendar months were grouped into two seasons: rainy (May–October) and dry (November–April). For temporal analysis, the study period was divided into three intervals (2011–2014, 2015–2019, and 2020–2024) to reflect major phases of malaria control in Burkina Faso. These periods correspond to the introduction of SMC in 2014, its nationwide scale-up by 2016, and the subsequent period of sustained implementation of malaria control interventions, including periodic LLIN distribution campaigns and the expansion of SMC to five cycles from 2023.

Proportions were compared using the chi-square (χ^2^) test, with statistical significance set at *p* < 0.05. Median age and measures of dispersion (minimum and maximum) were calculated after assessment of data normality. The geometric mean densities of trophozoites and gametocytes were calculated, along with their corresponding 95% confidence intervals (95% CI). Geometric mean trophozoite densities were compared across study years and predefined study periods using the Kruskal–Wallis test.

Malaria prevalence and corresponding 95% CI were estimated. Factors associated with malaria were assessed using univariate logistic regression, followed by multivariable logistic regression with a forward stepwise approach, including variables with *p* < 0.20 in univariate analysis. Odds ratios (ORs) and their 95% CIs were reported, and variables with 95% CIs not including 1 were considered significantly associated with malaria. All statistical tests were two-sided and considered statistically significant at *p* < 0.05.

### 2.7 Ethical considerations

This study used laboratory data only, involved no direct patient contact, and posed no physical or psychological risk. Data were handled anonymously and confidentially. Authorization to access laboratory registers was obtained from the Centre MURAZ. The study received ethical approval from the Comité d'éthique national pour la recherche en santé (approval number: 2024–042/MSHP/MESRI/CERS).

## 3 Results

### 3.1 General characteristics of data collected

Over the study period, 17,214 patients underwent both thick and thin blood smear examinations at the Parasitology–Mycology Laboratory of Centre MURAZ. The laboratory registers used for this study allowed the collection and analysis of several variables, although some information was incomplete. Sex data were available for 16,644 participants, while age data were recorded for 12,986 participants. Results of the thick and thin blood smear examination were documented for 17,044 participants. Among these, sex information was available for 16,475 individuals, age for 12,864, and complete data for all three variables (sex, age, and blood smear result) for 12,848 participants (Fig. 1).

**Fig. 1:**
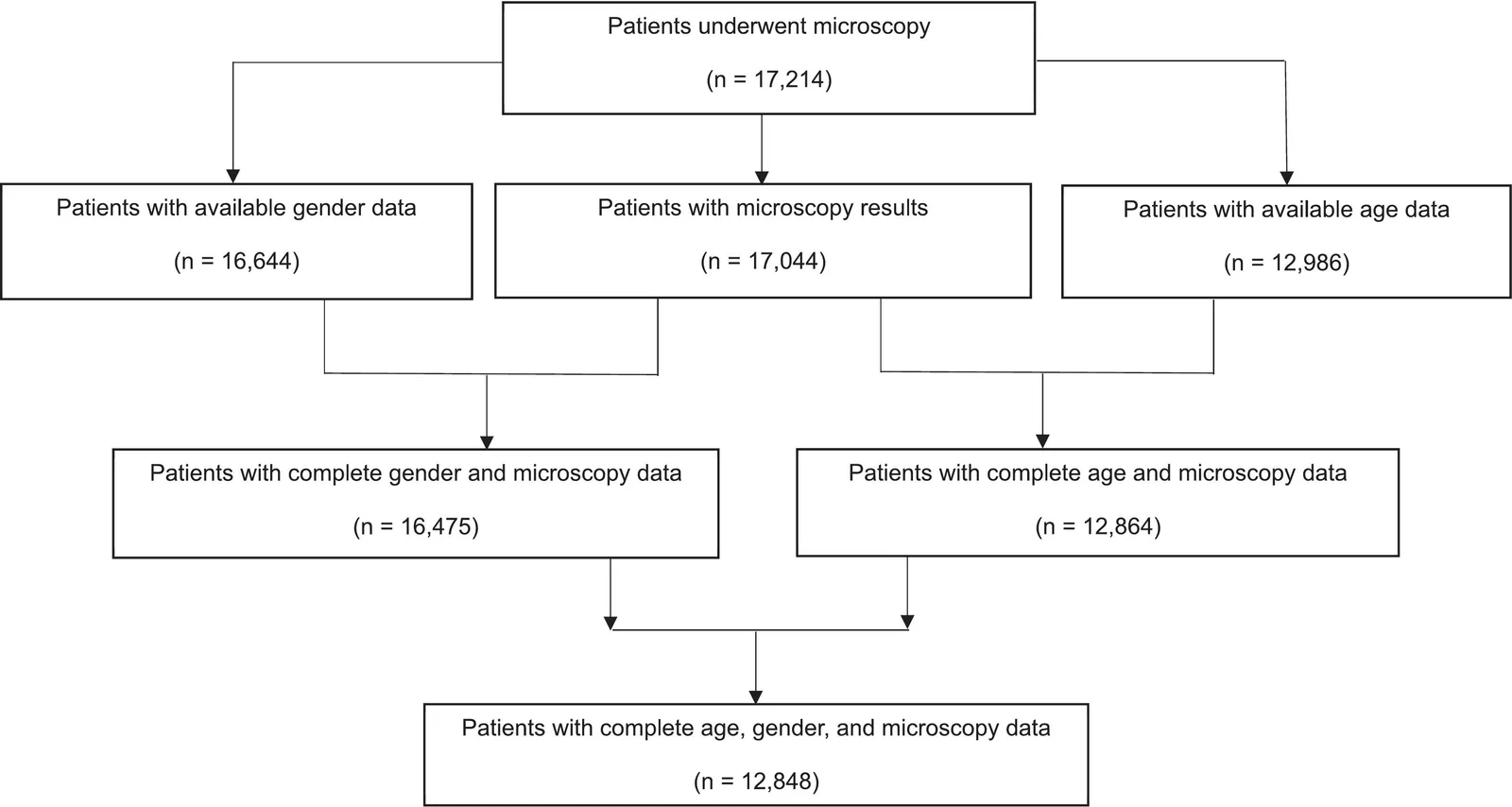
Flowchart of patients' enrolment for data analysis.

Among the 16,644 participants with available sex information, males accounted for 45.25% and females for 54.75%, with a gender ratio (M/F) of 0.83. Age information was available for 12,986 participants, covering all age groups, with a median age of 32 years (Minimum-Maximum: 1–97 years). Participants older than 26 years constituted the majority of the sample (61.26%), followed by those aged 16–26 years, who represented 18.68% of the total.

### 3.2 Malaria prevalence and plasmodium species detected

Among the 17,044 blood smear examinations with available results, *Plasmodium* spp. infections were detected in 2019 participants, yielding an overall malaria prevalence of 11.85%. The identified species were *P. falciparum*, *P. malariae*, and *P. ovale*, occurring as single or mixed infections. *P. falciparum* was the predominant species (97.52%), followed by *P. malariae* (1.14%) and *P. ovale* (0.84%). Mixed-species infections were uncommon, with *P. falciparum* + *P. malariae* and *P. falciparum* + *P. ovale* representing 0.30% and 0.20% of infections, respectively (Fig. 2).

**Fig. 2:**
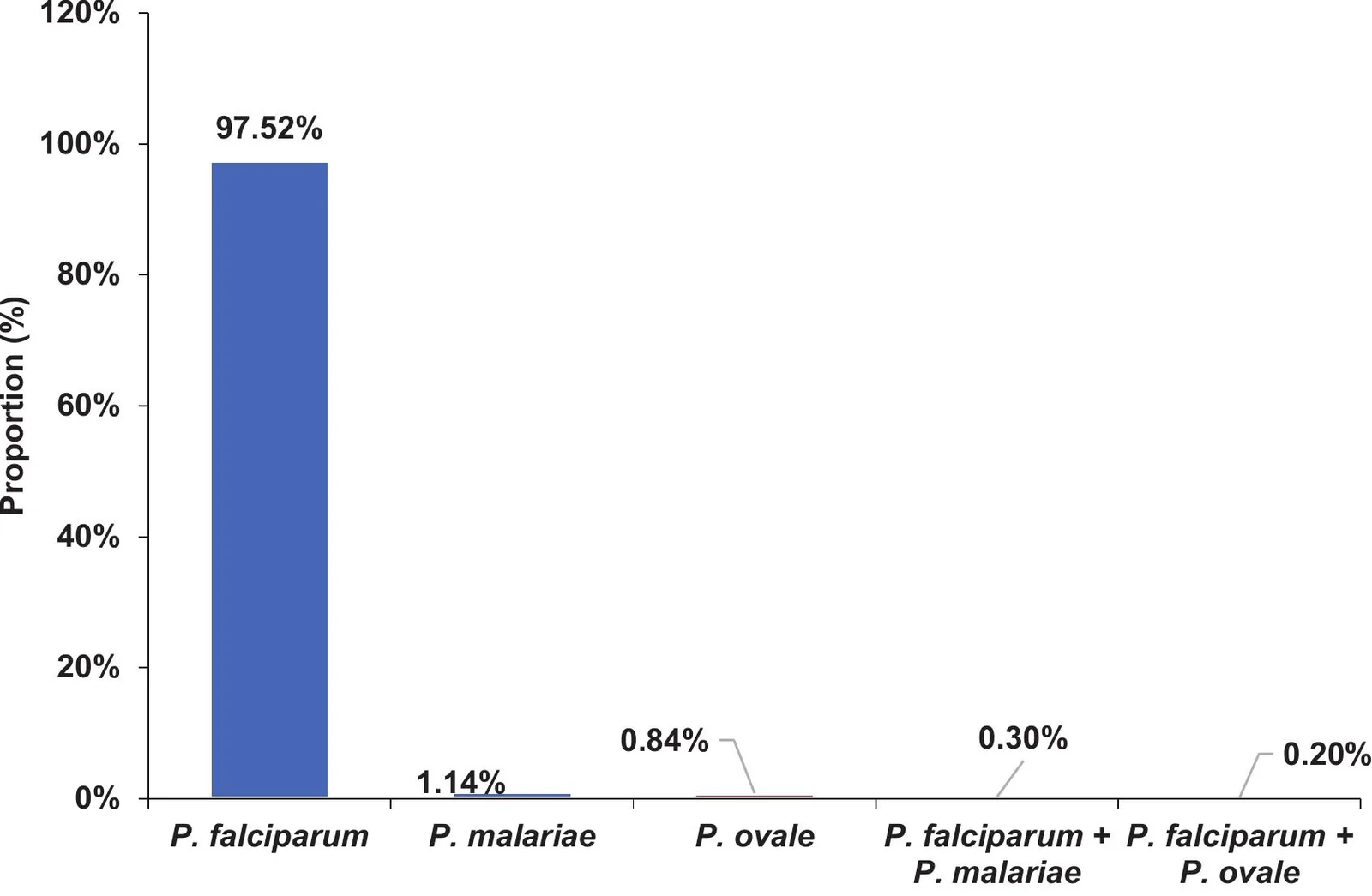
Distribution of *Plasmodium* species. P. falciparum: Plasmodium falciparum; P. malariae: Plasmodium malariae; *P. ovale*: Plasmodium ovale.

In positive samples, trophozoite stages were identified in 1998 individuals, gametocytes in 47 cases, and schizonts of *P. malariae* in a single case. The geometric mean densities of trophozoites and gametocytes were 786 parasites/μL (95% CI: 710.37–869.70) and 165.28 parasites/μL (95% CI: 101.71–268.60), respectively. The highest geometric mean trophozoite density was observed in 2012, at 5289.42 parasites/μL (95% CI: 3724.26–7512.37), whereas the lowest was recorded in 2020, at 227.47 parasites/μL (95% CI: 150.45–343.90) (Fig. 3). Trophozoite density varied significantly across study years (Kruskal–Wallis test, *p* < 0.001), with generally lower densities observed in the later years of the study. Consistent with this pattern, geometric mean trophozoite densities differed significantly across the predefined study periods (Kruskal–Wallis test, *p* < 0.001), decreasing from 3282.86 parasites/μL (95% CI: 2640.88–4080.00) in 2011–2014 to 670.85 parasites/μL (95% CI: 547.54–821.91) in 2015–2019 and 441.54 parasites/μL (95% CI: 393.99–494.81) in 2020–2024.

**Fig. 3:**
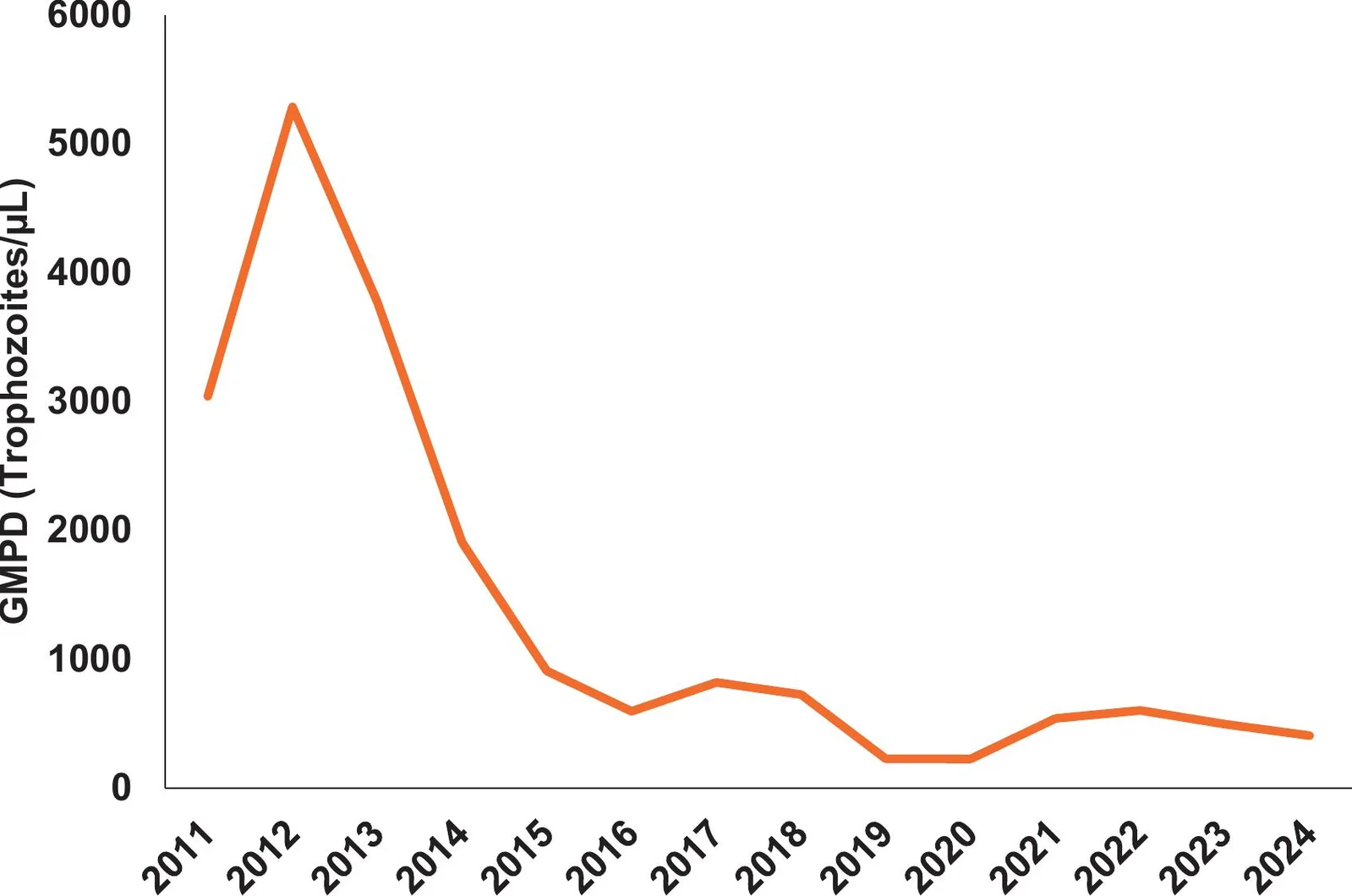
Yearly trends in geometric mean trophozoite densities. GMPD: Geometric Mean Parasite Density.

### 3.3 Yearly trends in malaria prevalence

Following a period of low prevalence between 2011 and 2015 (with a minimum of 1.85% in 2015), a gradual increase began in 2016, reaching a first peak in 2021 (22.28%). A slight decline was observed in 2022 (16.07%) and 2023 (17.20%), followed by a second peak in 2024 (31.26%), representing the highest value recorded during the study period. The year-to-year variation in prevalence was statistically significant according to the chi-square test for differences in proportions (*p* < 0.001) (Fig. 4).

**Fig. 4:**
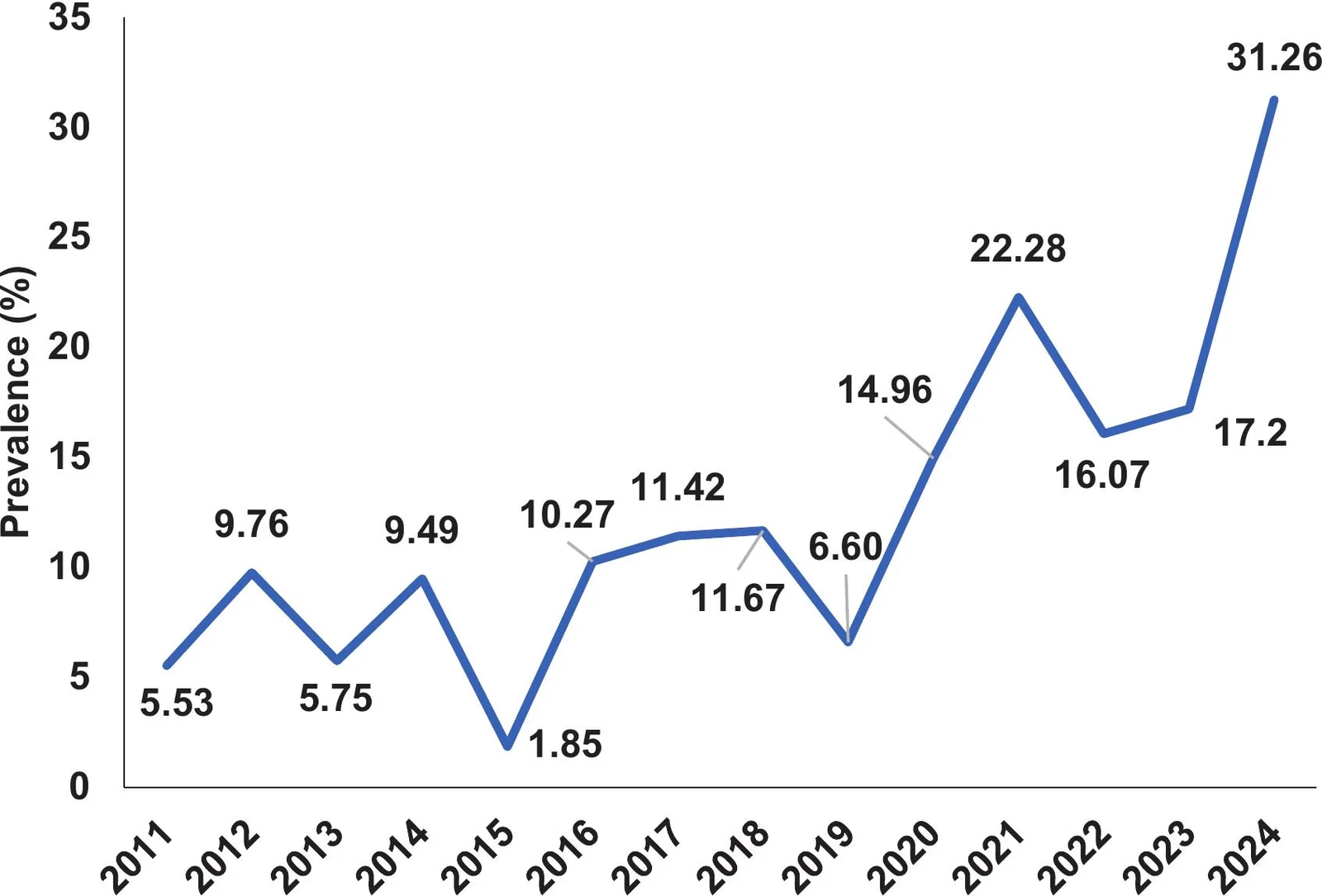
Yearly trends in malaria prevalence.

### 3.4 Monthly trends in malaria prevalence

Malaria was detected throughout the year, with prevalence increasing from July, peaking in October (22.52%), and declining through December. The highest prevalences were observed in September (18.42%), October (22.52%), and November (20.00%), whereas the lowest prevalence occurred in February (3.36%) (Fig. 5). A similar seasonal pattern was observed across the three study periods, with malaria prevalence increasing during the rainy season and reaching its highest levels between September and November (Supplementary File S1: Fig. S1). Monthly variations in prevalence were statistically significant according to the chi-square test for differences in proportions (*p* < 0.001).

**Fig. 5:**
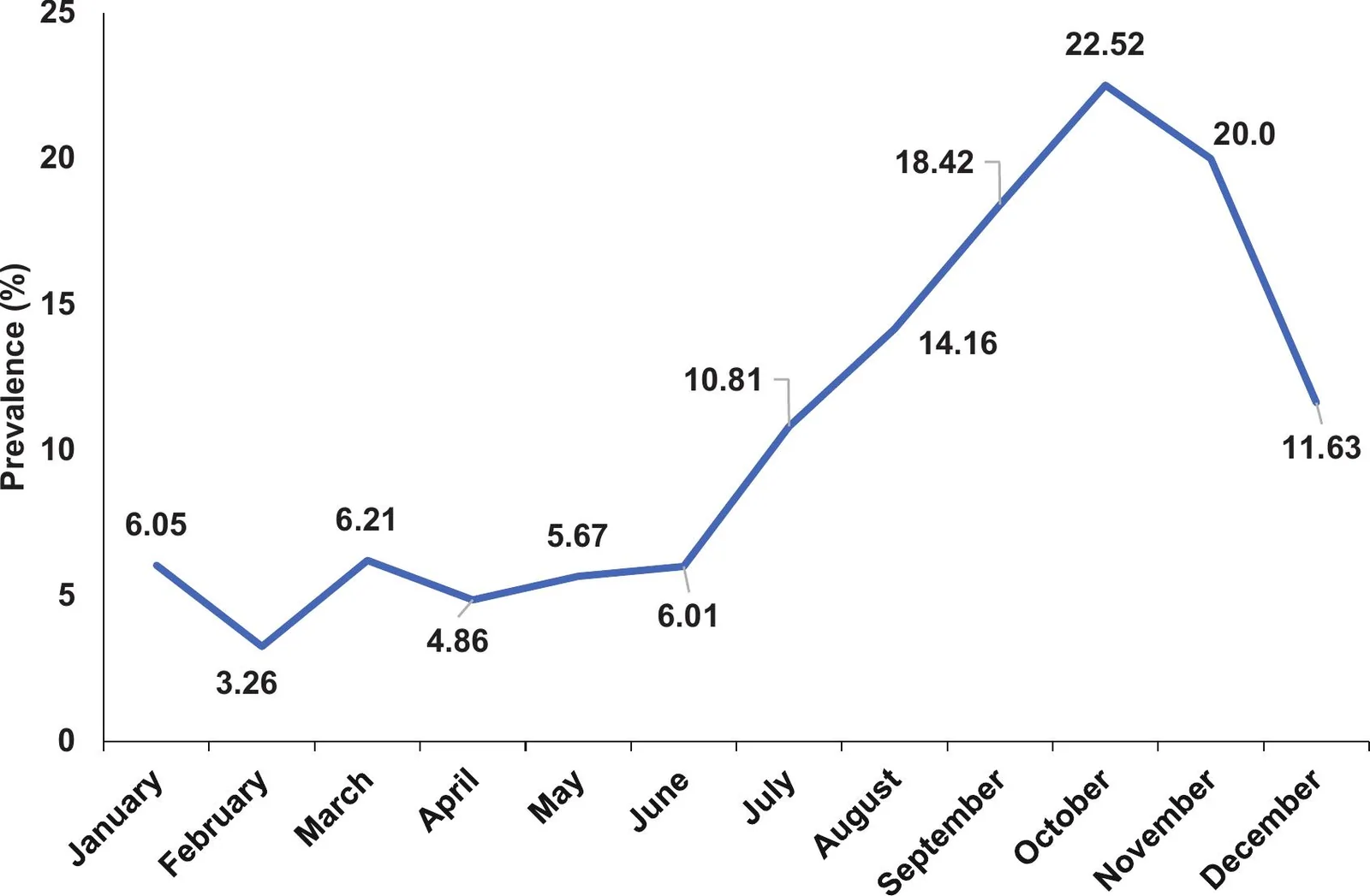
Monthly trends in malaria prevalence.

### 3.5 Risk factors for malaria

Univariate logistic regression, higher odds of malaria were observed during 2015–2019 (crude OR = 1.28; 95% CI: 1.12–1.44) and 2020–2024 (crude OR = 3.12; 95% CI: 2.77–3.52) compared with 2011–2014. The odds of malaria were also higher among individuals examined during the rainy season (crude OR = 1.68; 95% CI: 1.53–1.85), males (crude OR = 1.23; 95% CI: 1.12–1.36), and participants aged 5–15 years (crude OR = 1.54; 95% CI: 1.34–1.78) and 16–26 years (crude OR = 1.34; 95% CI: 1.17–1.53), compared with their respective reference groups (Table 1). Conversely, children aged <5 years had lower odds of malaria.

**Table 1:** Univariate and multivariate analyses of factors associated with malaria among study participants.

Variables	Malaria		COR (95% CI)	*P* value	AOR (95% CI)	*P* value
	Number tested	Number positive (%)				
**Study period**						
2011–2014	6004	460 (07.66)	1	–	1	–
2015–2019	6451	615 (09.53)	1.27 (1.12–1.44)	< 0.001	1.18 (1.02–1.34)	0.028
2020–2024	4589	944 (20.57)	3.12 (2.77–3.52)	< 0.001	3.02 (2.64–3.46)	< 0.001
**Season**						
Rainy	9008	1291 (14.33)	1.68 (1.53–1.85)	< 0.001	1.73 (1.55–1.93)	< 0.001
Dry	8036	728 (9.06)	1	–	1	–
**Sex**						
Female	9014	989 (10.97)	1	–	1	–
Male	7461	985 (13.20)	1.23 (1.12–1.36)	< 0.001	1.29 (1.16–1.43)	< 0.001
**Age group (years)**						
< 5*	885	73 (8.45)	0.69 (0.54–0.89)	0.004	–	–
5–15	1703	285 (16.74)	1.54 (1.34–1.78)	< 0.001	1.81 (1.56–2.10)	< 0.001
16–26	2395	355 (14.82)	1.34 (1.17–1.53)	< 0.001	1.50 (1.31–1.71)	< 0.001
˃ 26	7881	908 (12.60)	1	–	1	–

AOR: adjusted odds ratio; COR: crude odds ratio; *AOR not reported for <5 years because this category was not retained in the final model.

In the multivariable analysis, compared with 2011–2014, the odds of malaria were higher during 2015–2019 (aOR = 1.18; 95% CI: 1.02–1.34) and substantially higher during 2020–2024 (aOR = 3.02; 95% CI: 2.64–3.46). Compared with individuals examined during the dry season, those examined during the rainy season had higher odds of malaria (aOR = 1.73; 95% CI: 1.55–1.93). Similarly, males had higher odds of malaria than females (aOR = 1.29; 95% CI: 1.16–1.43). Participants aged 5–15 years (aOR = 1.81; 95% CI: 1.56–2.10) and 16–26 years (aOR = 1.50; 95% CI: 1.31–1.71) had higher odds of malaria than those aged >26 years (Table 1).

## 4 Discussion

The overall urban malaria prevalence observed in our study (11.85%) is consistent with estimates reported across Africa, where prevalence varies widely depending on epidemiological, environmental, and methodological contexts. In East Africa, particularly in Ethiopia, urban malaria prevalence has been reported to range from 7.5% to 32.6% (Alelign et al., 2018; Feleke et al., 2018; Gebretsadik et al., 2018; Shiferawu and Desta, 2021; Tesfaye and Teshome, 2022). Our findings fall within this range and are comparable to those reported in Ethiopian urban settings. In contrast, much higher prevalence has been reported in rural Central African settings, such as Cameroon, where Djoufounna et al. documented a prevalence of 65.3% between 2016 and 2020, reflecting environmental conditions more conducive to sustained vector proliferation (Djoufounna et al., 2024).

The malaria prevalence observed in the present study is lower than that reported in a previous study from Bobo-Dioulasso (Sirima, 2013) and in Senegal (Diallo et al., 2020), but remains consistent with the moderate yet persistent pattern of urban malaria transmission in West Africa. However, this overall prevalence conceals marked temporal variability. Malaria prevalence remained low during the early years of the study period, followed by a sustained increase from 2016 onward, with pronounced peaks in 2021 and 2024. These temporal patterns are in line with the results of the multivariable analysis, which identified year of diagnosis as an independent factor associated with malaria, with progressively higher risk observed in more recent periods, particularly during 2020–2024.

The gradual increase in malaria prevalence observed from 2016 onward may reflect the combined effects of rapid urban expansion, environmental changes favoring mosquito breeding, climatic variability, and challenges to malaria control (Merga et al., 2025). The peak observed in 2021 coincided with the COVID-19 pandemic, during which disruptions to healthcare services and malaria control activities were reported in several endemic settings (Benjamin et al., 2023). The even higher prevalence recorded in 2024 suggests that the increase in malaria transmission was not limited to the pandemic period but may represent a continuation of a broader upward trend. This pattern could be influenced by persistent environmental and demographic changes, as well as reduced effectiveness of malaria control interventions associated with increasing insecticide resistance in *Anopheles* vectors (Dabiré et al., 2012) and emerging antimalarial drug resistance in *Plasmodium* parasites (Tarama et al., 2023; Roh et al., 2023). However, these factors were not directly assessed in the present study, and their contribution to the observed trends cannot be established. Further investigations are needed to identify the drivers of the recent increase in malaria prevalence in Bobo-Dioulasso.

Seasonal analysis further supported these observations, with significantly higher odds of infection during the rainy season. A similar seasonal pattern was observed across the three study periods, with malaria prevalence generally increasing during the rainy season and reaching its highest levels during the late rainy season. This pattern is consistent with the increase in rainfall and the proliferation of breeding sites favorable to *Anopheles* mosquitoes. The persistence of low-level transmission during the dry season suggests the presence of permanent breeding sites capable of sustaining transmission year-round and seeding subsequent outbreaks, underscoring the importance of sustained surveillance in urban settings.

In the current study, analysis of *Plasmodium* species distribution showed a marked predominance of *P. falciparum*, accounting for 97.52% of infections, a finding consistent with reports from Bobo-Dioulasso and other urban settings in West Africa, including Dakar, Senegal (Sirima, 2013; Diallo et al., 2020). Despite this similarity, differences in local eco-epidemiological conditions should be considered when making comparisons. This finding suggests relative stability of the parasitological profile in urban Burkina Faso. The dominance of *P. falciparum* may be explained by its biological traits, adaptation to local vectors (Dabiré et al., 2012), and favorable environmental conditions, in addition to the widespread Duffy-negative phenotype in West African populations (Poirier et al., 2016; Brown et al., 2021), which constrains *P. vivax* circulation. However, reliance on routine microscopy and the retrospective design may have reduced sensitivity for detecting low-density or mixed infections, potentially leading to underestimation of non—*P. falciparum* species. These observations highlight the need for continued epidemiological surveillance of *Plasmodium* species distribution in urban African settings.

Regarding individual risk factors, male sex was identified as significantly associated with malaria in this study, consistent with findings from Burkina Faso, Senegal, and Ethiopia (Sirima, 2013; Alelign et al., 2018; Diallo et al., 2020; Tarekegn et al., 2021; File et al., 2019; Boresa et al., 2024). This association may reflect a combination of behavioral factors, including increased outdoor exposure during peak mosquito biting times and lower adherence to preventive measures, as well as as well as host-related biological factors, such as differences in body odor composition, skin microbiota, carbon dioxide production, and body temperature, which may influence mosquito attraction (Giraldo et al., 2023). However, contrasting patterns have been reported in other settings, such as rural Cameroon, where higher malaria risk among women has been attributed to agricultural activities, water-related domestic tasks, and pregnancy-associated vulnerability (Djoufounna et al., 2024). These differences likely reflect variations in local epidemiological contexts, transmission intensity, study design, and access to healthcare rather than true biological inconsistencies. Overall, these observations highlight the context-dependent nature of sex-related malaria risk and underscore the importance of routinely reporting sex-disaggregated data to better characterize malaria transmission patterns in urban settings.

In our study, age-stratified analysis revealed an atypical risk profile, with the highest risk observed among individuals aged 5–15 years and 16–26 years, while children under five years were less affected. This pattern differs from that observed in high-transmission rural settings but aligns with reports from several African urban contexts, where older children and young adults increasingly contribute to the malaria burden (Sirima, 2013; Alelign et al., 2018; Diallo et al., 2020; Tarekegn et al., 2021; File et al., 2019; Boresa et al., 2024; Moltot et al., 2024). The lower risk observed among young children may reflect the impact of targeted malaria control interventions in this age group. However, further analyses are needed to determine whether age-specific malaria risk changed across the different study periods. In contrast, the higher risk among school-aged children and young adults may be related to greater behavioral exposure, mobility, incomplete or waning immunity, and absence of targeted preventive measures. Taken together, these findings suggest a shift in the epidemiological profile of malaria in urban settings and highlight the importance of continued monitoring and further investigation to better understand the drivers of malaria transmission among older age groups.

This study has several limitations. First, its retrospective design relied on routinely collected laboratory data, with some missing information that may have affected statistical power. Second, malaria diagnosis was based on light microscopy, which may underestimate low-density or mixed infections. Third, analyses were restricted to symptomatic individuals, likely resulting in an underestimation of malaria prevalence at the population level. Fourth, the absence of data on key epidemiological factors and antimalarial resistance limited the assessment of risk determinants. In addition, although temporal variations in malaria prevalence were examined across three study periods, stratified analyses were not performed to determine whether associations with age, sex, and season differed between periods. Finally, as the study was conducted in a single urban setting and laboratory, the generalizability of the findings to other settings should be interpreted with caution.

## 5 Conclusion

This study documents a moderate malaria prevalence in Bobo-Dioulasso, predominantly due to *P. falciparum*, with marked seasonality peaking at the end of the rainy season. The increase in observed prevalence between 2011 and 2024 suggests evolving urban malaria transmission dynamics. Higher risk among males and individuals aged 5–26 years indicates a potential shift in the epidemiological profile. These findings highlight the value of continued surveillance and further evaluation of factors influencing malaria transmission in urban settings.

The following are the supplementary data related to this article.

Supplementary Fig. S1 Monthly trends in malaria prevalence according to the three study periods.

## CRediT authorship contribution statement

**Mamoudou Cissé:** Writing – review & editing, Writing – original draft, Visualization, Validation, Supervision, Methodology, Investigation, Formal analysis, Data curation, Conceptualization. **Kiswendsida Thierry Guiguemdé:** Writing – review & editing. **Issaka Zongo:** Writing – review & editing. **Nwintièro Léandre Gilchrist Gané Dabiré:** Writing – review & editing, Investigation. **Alamissa Soulama:** Writing – review & editing. **Somkéta Thomas Venceslas Sawadogo:** Writing – review & editing. **Seydou Nakanabo Diallo:** Writing – review & editing. **Adama Zida:** Writing – review & editing, Methodology.

## Ethical approval and consent to participate

This study was approved by the Comité d’éthique national pour la recherche en santé (approval number: 2024–042/MSHP/MESRI/CERS).

## Declaration of competing interest

The authors declare that they have no known competing financial interests or personal relationships that could have appeared to influence the work reported in this paper.

## Data Availability

Data is contained within the article.
