# Supplementary material for: Malaria trends in Bobo-Dioulasso, Burkina Faso (2011–2024): a retrospective laboratory-based study with implications for malaria control
Source: Parasite Epidemiol Control. 2026 Oct 1;35:e00551. doi: 10.1016/j.parepi.2026.e00551 (PMC13670388; doi:10.1016/j.parepi.2026.e00551)
Supplement: Supplementary file 1 [file mmc1.docx]

Prevalence (%)

|  | 2011-2014 | 2015-2019 | 2020-2024 |
| --- | --- | --- | --- |
| January | 4.43 | 4.73 | 11.35 |
| February | 0.42 | 4.35 | 5.2 |
| March | 1.69 | 4.75 | 15.96 |
| April | 1.93 | 4.63 | 9.54 |
| May | 2.45 | 4.11 | 15.11 |
| June | 5.39 | 4.76 | 10.53 |
| July | 5.53 | 9.49 | 21.21 |
| August | 9.78 | 9.24 | 27.06 |
| September | 11.9 | 15.02 | 27.75 |
| October | 18.44 | 21.07 | 27.75 |
| November | 14.72 | 16.03 | 30.41 |
| December | 7.18 | 11.49 | 16.21 |
